# Current Concept and Management of Spastic Hip in Children: A Narrative Review

**DOI:** 10.7759/cureus.43347

**Published:** 2023-08-11

**Authors:** Mohammed H Al-Rumaih, Mark W Camp, Unni G Narayanan

**Affiliations:** 1 Department of Orthopaedic Surgery, Prince Sultan Military Medical City, Riyadh, SAU; 2 Scientific Research Center (SRC), Prince Sultan Military Medical City, Riyadh, SAU; 3 Division of Orthopaedic Surgery, The Hospital for Sick Children, Toronto, CAN; 4 Department of Surgery, University of Toronto, Toronto, CAN; 5 Peter Gilgan Centre for Research and Learning, The Hospital for Sick Children, Toronto, CAN; 6 Bloorview Research Institute, Holland Bloorview Kids Rehabilitation Hospital, Toronto, CAN

**Keywords:** hip surveillance, surgical interventions, botulinum toxin, cerebral palsy, spastic hip disease

## Abstract

Cerebral palsy (CP) is a non-progressive motor condition that hinders the development of movement and posture. One of the common problems faced in CP is spastic hips, which can cause discomfort, deformity, and functional restrictions. This review article seeks to offer a thorough summary of the most recent methods for treating spastic hips in cerebral palsy patients. Additionally, it describes the success and potential risks of various conservative and surgical procedures. It also looks at new treatments and potential avenues for managing this complicated ailment.

## Introduction and background

Cerebral palsy (CP) is a neurological condition that affects the development of a large number of children around the world. Among the various manifestations of CP, spasticity in the hip joint poses significant challenges in terms of management and overall quality of life for affected individuals [1]. The spastic hip can lead to pain, deformities, and limitations in mobility, which can further impede activities of daily living and participation in society [2]. The spastic hip seen in cerebral palsy has been the focus of several therapeutic efforts throughout the years, ranging from conservative medicine to surgical correction. However, studies and discussions on the best methods of management continue. The purpose of this article is to provide a thorough and up-to-date analysis of the present state of knowledge concerning the treatment of spastic hips in people who have cerebral palsy.

## Review

Etiology and pathophysiology

Spastic hip in cerebral palsy is caused by injury or abnormality of the developing brain, which typically manifests in infancy or early childhood [3]. Hip spasticity can indeed be a consequence of cerebral palsy, but it can also arise from various other perinatal and postnatal causes like birth asphyxia, preterm birth, brain damage, infections, or strokes [4]. When the connections between the brain and the muscles are disrupted, the result is a spastic hip, characterized by aberrant muscle tone and hyperactive reflexes [5]. The primary motor cortex and associated neural circuits fail to regulate muscle activity effectively, leading to involuntary and sustained contractions of the muscles around the hip joint. The hip’s biomechanics and range of motion become impaired and stiffer as a result. Over time, the spasticity may cause deformities and structural changes in the hip joint, such as subluxation (partial dislocation) or dislocation, as well as the development of contractures [5]. The muscle imbalances can lead to abnormal forces on the hip joint, causing further joint degeneration and functional impairment.

Assessment and diagnosis

Cerebral palsy patients with spastic hips should have a thorough evaluation and diagnosis to determine the full scope of their problem and how it affects their daily functioning. There are often several stages.

Medical History

Collecting a thorough medical history is crucial for diagnosing and treating cerebral palsy and hip problems since it provides insight into the patient’s overall health, developmental milestones, and past interventions [6].

Physical Examination

It is essential to perform a complete physical examination of the hip. This includes assessing the range of motion, muscle tone, and any signs of spasticity, contractures, or dislocations [7].

Radiological Imaging

X-rays and other imaging techniques (e.g., MRI or CT scan) may be used to assess the alignment of the hip joint, detect any abnormalities, and determine the degree of bone deformities or hip dislocation [8]. X-rays are commonly used for this purpose, providing valuable information about the hip's condition. However, due to the challenges and difficulties associated with performing MRI or CT scans on such children, these imaging techniques are indicated when specific concerns or complex cases arise. In general, X-rays serve as the initial imaging modality, while MRI or CT scans are reserved for cases where a more comprehensive evaluation of the hip joint and surrounding structures is necessary. Careful consideration and close collaboration between the healthcare team and parents or caregivers are crucial in determining the most appropriate imaging approach for children with CP.

Gait Analysis

Observing the patient’s gait can provide valuable insights into their functional limitations and reveal compensatory movements or imbalances caused by the spastic hip [9].

Spasticity Evaluation

The assessment of spasticity involves identifying the muscle groups affected and measuring the severity of spasticity using standardized scales, such as the Modified Ashworth Scale [10].

Functional Assessment

The assessment of a child with CP and a spastic hip should encompass evaluating the impact on the patient's quality of life. This assessment may include the use of standardized questionnaires or functional tests [11]. However, it is crucial to emphasize the significant role of the Gross Motor Function Classification System (GMFCS) in this assessment and planning process. GMFCS plays a vital role in understanding the child's functional abilities and limitations, particularly concerning their hip condition. By incorporating GMFCS into the assessment, healthcare professionals can gain valuable insights into the child's overall motor function and tailor treatment plans more effectively to improve their quality of life.

Multidisciplinary Team Evaluation

Collaboration between various healthcare professionals, such as orthopedic surgeons, physiotherapists, occupational therapists, and neurologists, is vital for a comprehensive evaluation and accurate diagnosis [12].

After all of the data has been collected, a diagnosis can be made that takes into account factors such as the patient’s level of spasticity, the existence or absence of contractures or dislocations, and the severity of any associated sequelae. [13]. This information forms the basis for developing an appropriate management plan tailored to the individual needs of the patient.

Hip surveillance

Cerebral palsy patients with spastic hips, in particular, benefit greatly from regular hip monitoring. This process involves frequent clinical and radiological evaluations to closely monitor the development of the hips and identify any irregularities at an early stage. By catching potential issues early on, healthcare professionals can intervene promptly, which may help prevent serious complications like hip dislocation. Physical examinations by medical professionals trained to evaluate hip motion, muscle tone, and overall hip health in people with cerebral palsy are a standard part of routine clinical assessments. X-rays and other forms of radiological analysis are used to capture high-resolution images of the hip joint for a more thorough analysis of the joint’s anatomy and alignment [14,15]. Hip surveillance’s ultimate aim is to track any changes in the hips over time, as spasticity and abnormal muscle tone in cerebral palsy can cause progressive alterations in hip development. With regular monitoring, healthcare providers can identify subtle signs of hip dysplasia, subluxation (partial dislocation), or dislocation. Early detection of such issues enables medical professionals to implement appropriate interventions to maintain or improve hip function and prevent long-term complications.

Management

Conservative Management

In order to enhance hip function and prevent further difficulties, conservative therapy of the spastic hip in cerebral palsy employs non-invasive techniques [16]. Physical therapy, stretching, orthotics, and other assistive technologies may be used [17]. The conservative management strategy relies heavily on physical therapy. Expert therapists help patients develop individualized workout plans that strengthen the hip-supporting muscles. These exercises aim to maintain and improve joint range of motion, strengthen weakened muscles, and promote overall mobility. Stretching exercises are also essential in managing spastic hips. By stretching the tight and overactive muscles, therapists help reduce muscle tightness and alleviate the discomfort caused by spasticity. Contractures are frequent in people with cerebral palsy, but they can be prevented with regular stretching regimens. These exercises should focus on the specific muscle groups involved, such as the hip flexors, adductors, and hamstrings. By incorporating exercises like hip flexor stretches, adductor stretches, and hamstring stretches, therapists can work to prevent contractures and maintain healthy joint mobility. Regular and consistent participation in these targeted stretching routines is instrumental in promoting flexibility, minimizing muscle tightness, and enhancing overall comfort and functionality for individuals with spastic hips. Orthotic devices, including specially designed braces or splints, may be prescribed to provide essential support and stability to the hip joint. These orthotic devices are carefully chosen based on the individual's needs and may encompass various types, such as hip abduction braces or lower limb splints. These devices are instrumental in not only maintaining optimal alignment but also mitigating the potential risk of deformities. By offering targeted support, it contributes to improved posture and enhanced functional capabilities for the individual. The choice of orthotic intervention is personalized, considering factors like the nature of the hip condition and the patient's overall mobility requirements. Additionally, assistive devices can be used to enhance mobility and independence. Devices like walkers or crutches can provide support while walking, enabling patients to engage in activities that might otherwise be challenging.

Botulinum Toxin Injections

Botulinum Toxin A (Botox) injections are used to temporarily weaken spastic muscles around the hip joint. Muscles such as the hip adductors, flexors, and abductors are commonly targeted for Botox treatment due to their involvement in hip spasticity. They work by blocking the release of a chemical messenger called acetylcholine, which is responsible for muscle contractions. This treatment can help relieve muscle tightness and improve joint alignment [18,19]. After administering Botox, a comprehensive management plan is typically implemented. This involves collaborating with rehabilitation specialists to optimize the benefits of muscle weakening. Physical therapy and stretching exercises play a pivotal role in maintaining joint range of motion, promoting muscle balance, and preventing contractures. Botox injections are often used as an adjunct to conservative management, allowing for better outcomes in some cases.

Surgical Interventions

Surgical interventions play a crucial role in addressing hip subluxation or dislocation when conservative treatments prove inadequate or when the condition becomes severe. In cases where non-surgical approaches do not yield satisfactory results, surgical procedures become necessary to improve joint stability and functional outcomes. A frequently employed surgical technique is soft tissue release, which specifically targets the commonly affected tight or contracted muscles and ligaments around the hip joint in cerebral palsy. For instance, the hip adductors and flexors are often the focus of this approach. In soft tissue releases, these muscles and ligaments are meticulously cut or lengthened through surgical procedures. This intervention is designed to significantly enhance joint mobility and effectively alleviate pressure on the hip. By precisely addressing these tight structures, the ultimate goal is to restore proper alignment and functional capability. The selection of muscles for release and the method used are based on a thorough assessment of the individual's condition and the expertise of the surgical team. This tailored approach ensures the best possible outcomes for the patient's unique hip spasticity profile [20]. Bony realignments encompass a range of specific surgical procedures aimed at correcting hip abnormalities in cerebral palsy. Among these procedures are Varus osteotomies, open reduction techniques, shortening osteotomies, and acetabular procedures. Through Varus osteotomies, the alignment of the femoral head and neck is adjusted to address hip subluxation. Open reduction techniques involve surgical repositioning of the femoral head within the hip socket, improving stability. Shortening osteotomies focus on adjusting the length of the femur to enhance alignment and reduce tension. Acetabular procedures center on reshaping the acetabulum, the hip socket, to promote optimal joint congruence. Bony realignments are particularly effective in cases where bone deformities play a significant role in hip subluxation or dislocation. By employing these specific procedures, surgical teams aim to not only improve alignment and stability but also enhance overall hip function for individuals with cerebral palsy. The choice of procedure depends on the patient's unique condition and the expert assessment of the surgical team. In more complex situations, hip reconstruction surgeries may be considered. Hip reconstruction involves comprehensive and specialized procedures that may include repositioning bones, repairing damaged cartilage, and addressing any other structural issues affecting the hip joint. The end result is meant to be a joint that is both more stable and functional, allowing for more range of motion and less discomfort [21]. It is important to note that surgical interventions are not standalone treatments; they are often combined with other therapeutic modalities to achieve the best outcomes. Post-surgery, rehabilitation and physical therapy are typically prescribed to aid in the recovery process and help the patient regain strength, flexibility, and function. The GMFCS level also plays a crucial role in determining the appropriate treatment approach. For higher GMFCS levels with more significant functional limitations, surgical interventions may be considered earlier to improve joint stability and functionality. However, surgical interventions are not standalone treatments; they are often combined with other therapeutic modalities for optimal results. Rehabilitation and physical therapy post-surgery help patients regain strength, flexibility, and function. Ultimately, the decision to pursue conservative management or surgery, as well as the choice between soft tissue or bony procedures, is based on a thorough evaluation of the individual's condition, their response to initial treatments, and the expertise and recommendations of the healthcare team.

Emerging therapies and novel approaches

In the field of cerebral palsy management, emerging therapies and novel approaches have been paving the way for more effective treatments for the spastic hip. Surgical procedures that can selectively target and block neural pathways responsible for spasticity, such as selective dorsal rhizotomy (SDR), have gained popularity in recent years [22]. Advances in orthopedic techniques have also contributed to improved treatment options. Minimally invasive surgery is one such advancement, which involves using smaller incisions and specialized tools to perform surgical procedures with reduced trauma and quicker recovery times. This approach has shown promise in treating spastic hips in cerebral palsy patients [23]. Additionally, the development of patient-specific implants has opened new possibilities for individualized and more effective treatments. These implants are tailored to a patient’s unique anatomy, ensuring a better fit and potentially leading to improved outcomes compared to generic implants [24]. New treatments and innovative methods give people with cerebral palsy who suffer from spastic hips hope for a brighter future and a higher quality of life. As research and technology continue to advance, we can anticipate even more innovative solutions in the future.

Rehabilitation and postoperative care

The care of the spastic hip in cerebral palsy is greatly aided by rehabilitation [25]. To improve muscular strength, joint range of motion, and functional abilities, physiotherapy and occupational therapy are essential parts of postoperative treatment [26]. To design effective postoperative rehabilitation programs, customized exercise plans are created to suit each patient’s specific needs and capabilities. These exercises are aimed at improving gait patterns and helping the individual achieve optimal mobility. Additionally, adaptive equipment may be introduced to assist in performing daily activities and promoting independence. A multidisciplinary strategy is essential for effective rehabilitation. This requires a multidisciplinary group of doctors, nurses, and therapists to offer each patient the individualized attention they need. By working together, the team is better able to assess the whole person, which improves the quality of care given to the patient as well as the speed and extent to which they recover.

Future directions

Future directions in the care of the spastic hip in cerebral palsy show promising promises as research and technology advance. Innovative treatments, such as regenerative therapies using stem cells or growth factors, may offer the potential to promote tissue repair and functional recovery [27]. To help enhance motor skills and gait in children with cerebral palsy, advances in robotic-assisted rehabilitation equipment and virtual reality-based therapy are predicted [28]. Moreover, ongoing research into neuroplasticity and neural modulation techniques may lead to non-invasive approaches for controlling spasticity and promoting better hip joint function.

## Conclusions

Spastic hips in cerebral palsy must be treated using a comprehensive and interdisciplinary approach. Early diagnosis and timely surgical correction of spastic hip displacement can prevent hip dislocation and the need for more extensive surgery. We advise building the screening program utilizing the GMFCS Level. Ongoing research and innovation will influence the future of spastic hip care, with the ultimate goal of improving the quality of life for people with cerebral palsy.
